# The Strengths and Obstacles in the Differential Diagnosis of Progressive Supranuclear Palsy—Parkinsonism Predominant (PSP-P) and Multiple System Atrophy (MSA) Using Magnetic Resonance Imaging (MRI) and Perfusion Single Photon Emission Computed Tomography (SPECT)

**DOI:** 10.3390/diagnostics12020385

**Published:** 2022-02-02

**Authors:** Piotr Alster, Michał Nieciecki, Bartosz Migda, Michał Kutyłowski, Natalia Madetko, Karolina Duszyńska-Wąs, Ingeborga Charzyńska, Dariusz Koziorowski, Leszek Królicki, Andrzej Friedman

**Affiliations:** 1 Department of Neurology, Medical University of Warsaw, 03-242 Warsaw, Poland; natalia.madetko@wum.edu.pl (N.M.); karolina.duszynska@gmail.com (K.D.-W.); dariusz.koziorowski@wum.edu.pl (D.K.); andrzej.friedman@wum.edu.pl (A.F.); 2 Department of Nuclear Medicine, Children’s Memorial Health Institute, 04-730 Warsaw, Poland; msnieciecki@gmail.com; 3 Diagnostic Ultrasound Lab, Department of Pediatric Radiology, Medical Faculty, Medical University of Warsaw, 03-242 Warsaw, Poland; bartoszmigda@gmail.com; 4 Department of Radiology, Mazovian Brodnowski Hospital, 03-242 Warsaw, Poland; michael.kutylowski@gmail.com; 5 Department of Nuclear Medicine, Mazovian Brodno Hospital, 03-242 Warsaw, Poland; ingach@gazeta.pl (I.C.); leszek.krolicki@wum.edu.pl (L.K.); 6 Department of Nuclear Medicine, Medical University of Warsaw, 02-097 Warsaw, Poland

**Keywords:** progressive supranuclear palsy, corticobasal syndrome, multiple system atrophy, atypical parkinsonism, SPECT, MRI, neuroimaging

## Abstract

Multiple System Atrophy—Parkinsonism Predominant (MSA-P) and Progressive Supranuclear Palsy—Parkinsonism Predominant (PSP-P) are the clinical manifestations of atypical parkinsonism. Currently, there are no efficient in vivo methods available relating to neuroimaging or biochemical analysis in the examination of these entities. Among the advanced methods available, using positron emission tomography is constrained by high cost and low accessibility. In this study the authors examined patients with two types of atypical parkinsonism—MSA-P and PSP-P, which are difficult to differentiate, especially in the early years of their development. The aim of this study was to assess whether the examination of patients in the period following the early years (3–6-year duration of symptoms) could be enhanced by perfusion single photon emission computed tomography (SPECT), magnetic resonance imaging (MRI) or evaluation of cognitive abilities. Extended examination using MRI and perfusion SPECT showed that the evaluation of the mesencephalon/pons ratio, mesencephalic volume decrease, the Magnetic Resonance Parkinsonism Index (MRPI) and frontal perfusion should be considered more feasible than screening cognitive evaluation in MSA-P and PSP-P with a 3–6-year duration of symptoms.

## 1. Introduction

The assessment of atypical parkinsonism remains a difficult issue. The only definite diagnosis of Progressive Supranuclear Palsy (PSP) and multiple system atrophy (MSA) is based on neuropathological evaluation [1,2,3]. The differential diagnosis of the common variants of PSP—PSP Richardson Syndrome (PSP-RS) and Multiple System Atrophy—Parkinsonian type (MSA-P) seems simple due to the characteristic symptoms; however, it becomes more difficult when PSP—Parkinsonism Predominant (PSP-P), the second most common phenotype, is assessed. Such factors as the presence of dysautonomia, Rapid Eye Movement (REM) behavior disorder (RBD) and preserved cognitive abilities may be present in both diseases [1,2,3]. The frequency of the presence of these symptoms differs between MSA-P and PSP-P [4]. Additionally, manifestations typically associated with the course of PSP-RS, such as oculomotor dysfunction, postural instability and poor response to levodopa treatment, may be absent in PSP-P even after a few years of disease duration [4]. This leads to a need for tools that are capable of making a differential diagnosis of the diseases. The current criteria used for the diagnosis of PSP and MSA highlight neuroimaging—magnetic resonance imaging (MRI) and single photon emission computed tomography (SPECT)—as an additional tool that possibly complements clinical examination [1,2,3]. The reliability of the additional testing is bound by its limited specificity. Neuroimaging in the examination of atypical parkinsonian syndromes was extensively described using MRI, SPECT, positron emission tomography (PET) and other less common methods. The atrophy within the mesencephalon previously described cannot always be properly examined due to fact that MRI, which enables its proper assessment, cannot be used on all patients. Other methods, such as verifying hyperechogenic substantia nigra in transcranial sonography, were negatively verified through the exploration of the issue of PSP-P in various studies [5,6,7,8,9,10,11]. The analyses of perfusion in atypical parkinsonism showed hypoperfusion in the cerebellum in MSA and in the thalamus in PSP [12]. However, the methods did not provide any tools which would introduce significant advances in differential diagnosis [12,13]. Combined dopamine transporter and perfusion SPECT showed differentiating potential in the examination of neurodegenerative diseases lacking clinical information [14]. PET examination using fluorodeoxyglucose did not present any significant advancement in the in vivo assessment when compared with the in vivo perfusion SPECT [15]. Tau radiotracers such as ^18^F-AV1451 are affected by off-binding affinity associated with neuromelanin or monoaminoxidase [16]. Second-generation radiotracers such as ^18^F-PI2620 provide a more specific analysis of tauopathic atypical parkinsonism, but limited access and the high cost of examination currently prevents the possible clinical use of the method [17]. Among other tools supporting clinical diagnosis of PSP, the Magnetic Resonance Parkinsonism Index (MRPI) should be mentioned. MRPI is calculated as the pons/midbrain width ratio multiplied by the middle cerebellar peduncles/superior cerebellar peduncles width ratio. As has been recently published, automated MRPI calculation by a web-based platform showed a high accuracy in indicating PSP-RS and PSP-P (93.6% and 86.5%, respectively), even in the early stages of the disease [18]. Some studies indicate the usefulness of parameters assessing cortical atrophy, especially in the frontal lobes, in PSP differentiation [15]. Numerous studies describe high sensitivity and specificity of multiple region assessment in APS differential diagnosis instead of focusing on only one parameter [18]. Additionally, the decrease in the width of the middle cerebellar peduncle (MCP) was reported in MSA [19,20]. The aim of this study was to assess the differentiating potential of MRI and SPECT in the assessment of atypical parkinsonisms showing possible resemblance, as is the case in PSP-P and MSA-P with a 3–6-year history of disease duration.

## 2. Materials

Thirty-six patients with atypical parkinsonism were included in the study: 16 patients (10 females, 6 males) with PSP-P, aged 61 to 81, and 20 patients (13 females, 7 males) with MSA-P, aged 50 to 81 (Table S1). The disease duration among all patients varied from 3 to 6 years. The clinical diagnosis was based on recent criteria of diagnosis of PSP and MSA [1,2,3]. Due to the fact that MSA-P was observed among younger patients, the groups could not be age-matched. All of the clinical examinations were performed by neurologists experienced in movement disorders from a single Department of Neurology. All of the patients included in the study were hospitalized at the Department of Neurology between November 2016 and December 2019. These patients were admitted to the Department of Neurology after an analysis of the Department of Neurology database; patients with a clinical diagnosis of PSP (the primary diagnosis in the database did not indicate different phenotypes of PSP) and MSA-P were selected. Among the patients in both groups, such features as moderate/weak response to levodopa treatment, dysautonomia and RBD were present. Patients with significant vascular changes were excluded from the study. The group with “significant vascular changes” included patients with lesions (detectable using the T2 sequences in MRI >1 mm) and patients who had suffered stroke or transient ischemic attack (TIA). The TIA group was also excluded due to the higher risk of possible small ischemic changes in the brain, which could possibly be undetectable by MRI. Additionally, patients with TIA have a significantly higher risk of experiencing a stroke, suggesting the need to avoid the inclusion of this group in the study. No patients were excluded during the course of the study. The study was approved by the local ethical committee.

## 3. Methods

### 3.1. MRI

Measurements concerning the third ventricle width, pons, midbrain area, pons/midbrain ratio, MRPI and the width of MCP and the superior cerebellar peduncle (SCP) were based on assessments made using MRI Siemens Skyra 3.0 Tesla. T2 sequences (sagittal T2, TR = 4420 ms, TE = 99 ms, FOV = 220 mm, resolution 320 × 320 and axial T2, TR = 4769 ms, TE = 96 ms, FOV = 220 mm, resolution 448 × 392) were used for anatomic measurement as they are part of a worldwide standard brain protocol and have commonly been used for morphometric brain studies by other investigators [19,20,21,22]. All measurements were performed manually by physicians with at least 5-years’ experience in diagnostic imaging. The width of the 3rd ventricle was measured as the maximum width of the 3rd ventricle in the axial plane [23,24]. The areas of the midbrain and the pons were measured, as proposed by Oba et al., on the mid-sagittal MRI using the display tools of a Siemens workstation [21,22,24]. The ratio of the area of the midbrain to the area of the pons was evaluated in all subjects. Due to the higher occurrence of abnormalities within MCP, additional measurements regarding the widths of the MCP and SCP were taken.

### 3.2. SPECT

The methodological aspect of SPECT was similar to that used in a previous study of the same group [25]. The radiotracer used to assess regional cerebral blood flow was technetium-99m hexamethylpropyleneamine oxime (^99m^Tc-HMPAO). An amount of 740 mBq of ^99m^Tc-HMPAO was administered to the patients in a quiet, dimly lit room. The acquisition was performed in a supine position with a SPECT/CT scan (Symbia T6, Siemens) on a dual-head gamma camera with a low-energy high-resolution parallel-hole collimator. A step-and-shoot acquisition mode was utilized. Sequences of 128 frames on a 128 × 128 matrix were used (64 projections per head, 30 s per projection). The photopeak was set at 140 keV with a 10% window on either side of it. Iterative reconstruction (eight iterations, eight subsets, 7 mm Gauss filter), scatter correction and CT attenuation correction were undertaken. Post-processing assessment was performed using Scenium software (Siemens Medical Solutions USA, Inc., Malvern, PA, USA). SPECT ROIs were pre-planned in Scenium software (an integral part of the Siemens workstation) based on T1 MRI images of a standard brain dataset. The shape and size of the investigated brains SPECT were adjusted to the shape and size of the standard brains from the dataset. The pre-planned ROIs were then extrapolated to the SPECT images of the investigated brains. Subsequently, total maximum and minimum counts were automatically measured in each ROI of investigated brain SPECT and were compared using Scenium with measurements from the standard brain SPECT datasets. All comparisons were automatically presented by Scenium as standard deviations. Values of standard deviations from ROIs were evaluated in multiple locations in the brain by statistical analysis.

### 3.3. Cognitive Screening

Cognitive status was assessed by two screening methods: Mini Mental State Examination (MMSE) and Montreal Cognitive Assessment (MoCA). Both MMSE and MoCA are widely known brief cognitive tests for the screening of cognitive impairment [26,27,28]. Validity of these methods in detecting cognitive impairment either in Parkinson’s Disease or atypical parkinsonism is well documented. However, the MoCA test is shown to have better sensitivity and specificity than the MMSE test. Each patient was examined by a neuropsychologist experienced in assessment of patients with atypical parkinsonisms.

## 4. Statistical Analysis

All analyses were performed using the Statistica software (version 13.1 Dell. Inc. Statsoft, Round Rock, TX, USA). The distribution of continuous data was assessed by the W Shapiro–Wilk test. Descriptive statistics are presented as mean values within range (minimal and maximal values) and standard deviations with a 95% confidence interval. Subgroup analysis was performed with a Student’s *t*-test. Significant *p* values are marked in red for easier reading. The significant difference for group comparison was set at the threshold *p* = 0.0019, after Bonferroni’s correction for multiple comparisons.

## 5. Results

All descriptive statistics for each disease entity are presented in Table 1 in relation to psychological tests, MRI and SPECT imaging. Authors used the more restrictive significance threshold after Bonferroni correction.

### 5.1. Subgroup Analysis

#### 5.1.1. Psychological Tests: MMSE and MoCA 

There was no significant difference in MMSE and MoCA results between patients with PSP-P and MSA-P (Table 1 and Table 2).

#### 5.1.2. MRI

**Figure 1:**
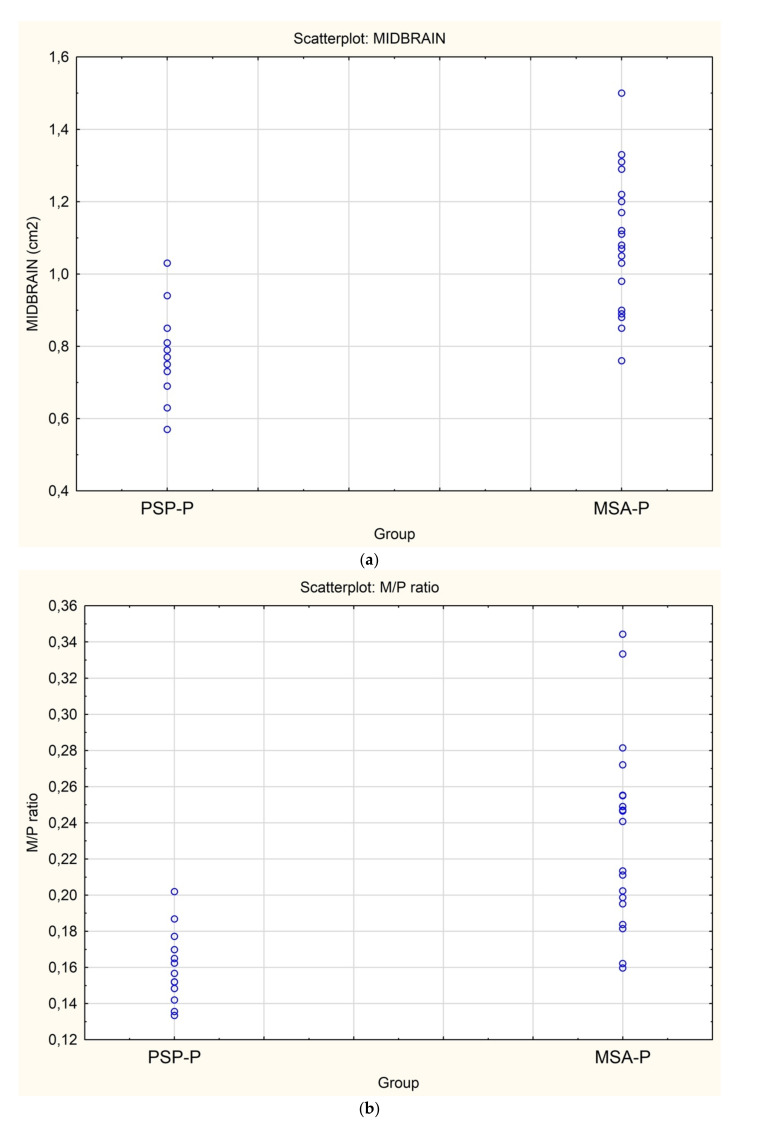

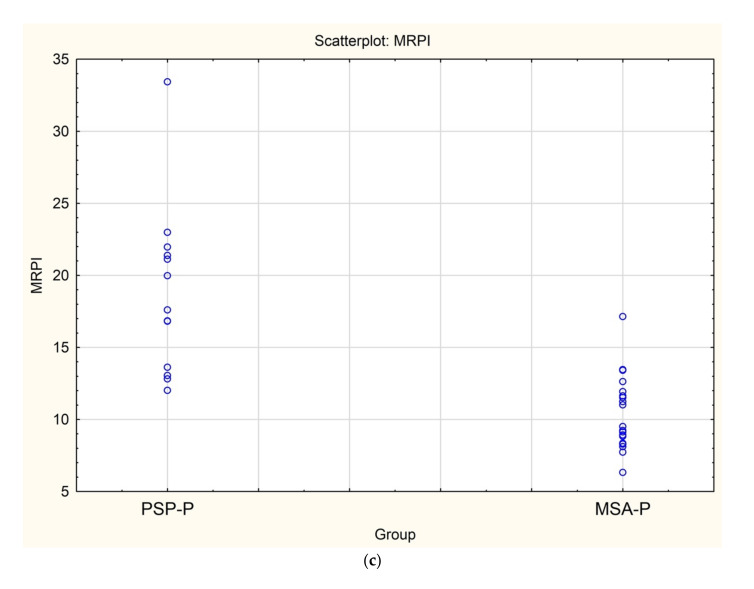

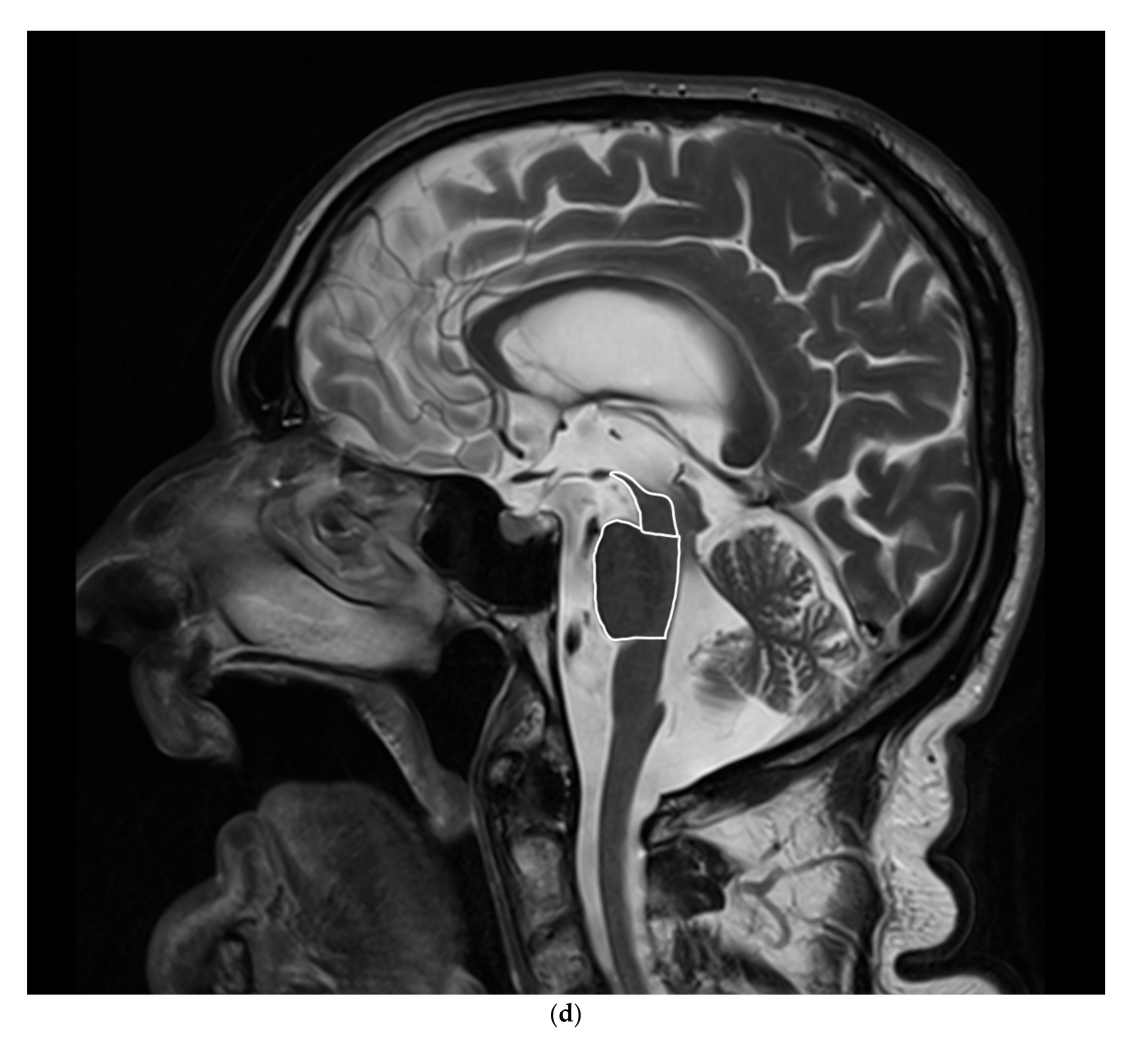
(**a**)**.** Scatterplot presenting the significant differences between MSA-P and PSP-P in the midbrain surface, (**b**) Scatterplot presenting the significant differences between MSA-P and PSP-P in the M/P ratio, (**c**) Scatterplot presenting the significant differences between MSA-P and PSP-P in the MRPI, (**d**) The atrophy of the mesencephalon in the MRI of a patient with PSP-P.

There was a significantly smaller midbrain surface in patients with PSP-P in comparison with patients with MSA–P, 0.76 cm^2^ vs. 1.08 cm^2^. Moreover, the ratio of midbrain to pons (M/P ratio) was also significantly smaller in patients with PSP-P vs. MSA-P 0.16 vs. 0.23, but the MRPI values were higher in patients with PSP-P vs. MSA-P 18.75 vs. 10.6 (Table 1 and Table 2) (Figure 1a–d).

#### 5.1.3. SPECT

**Figure 2:**
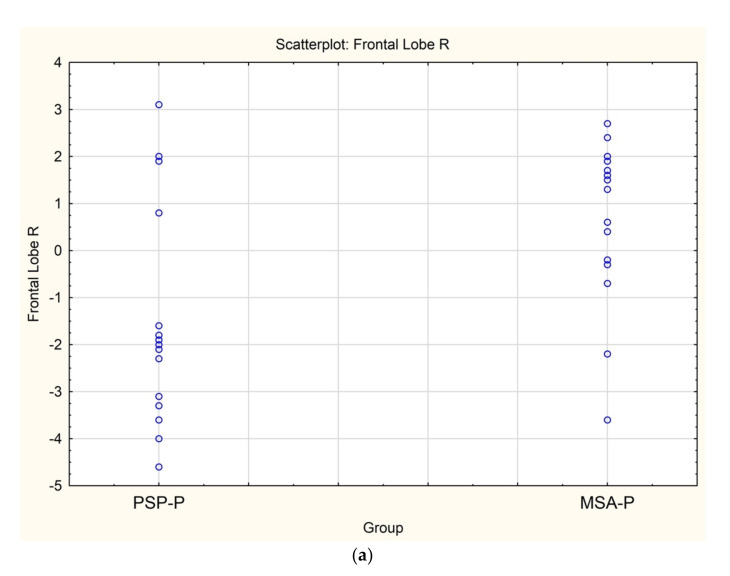

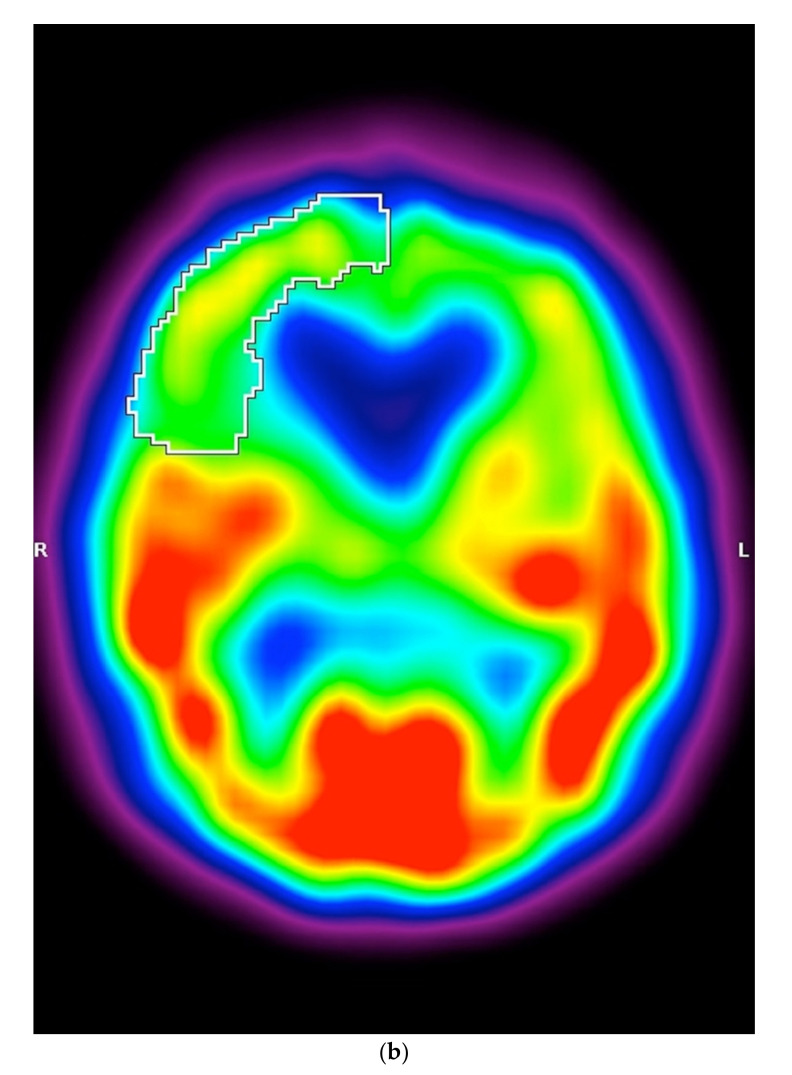

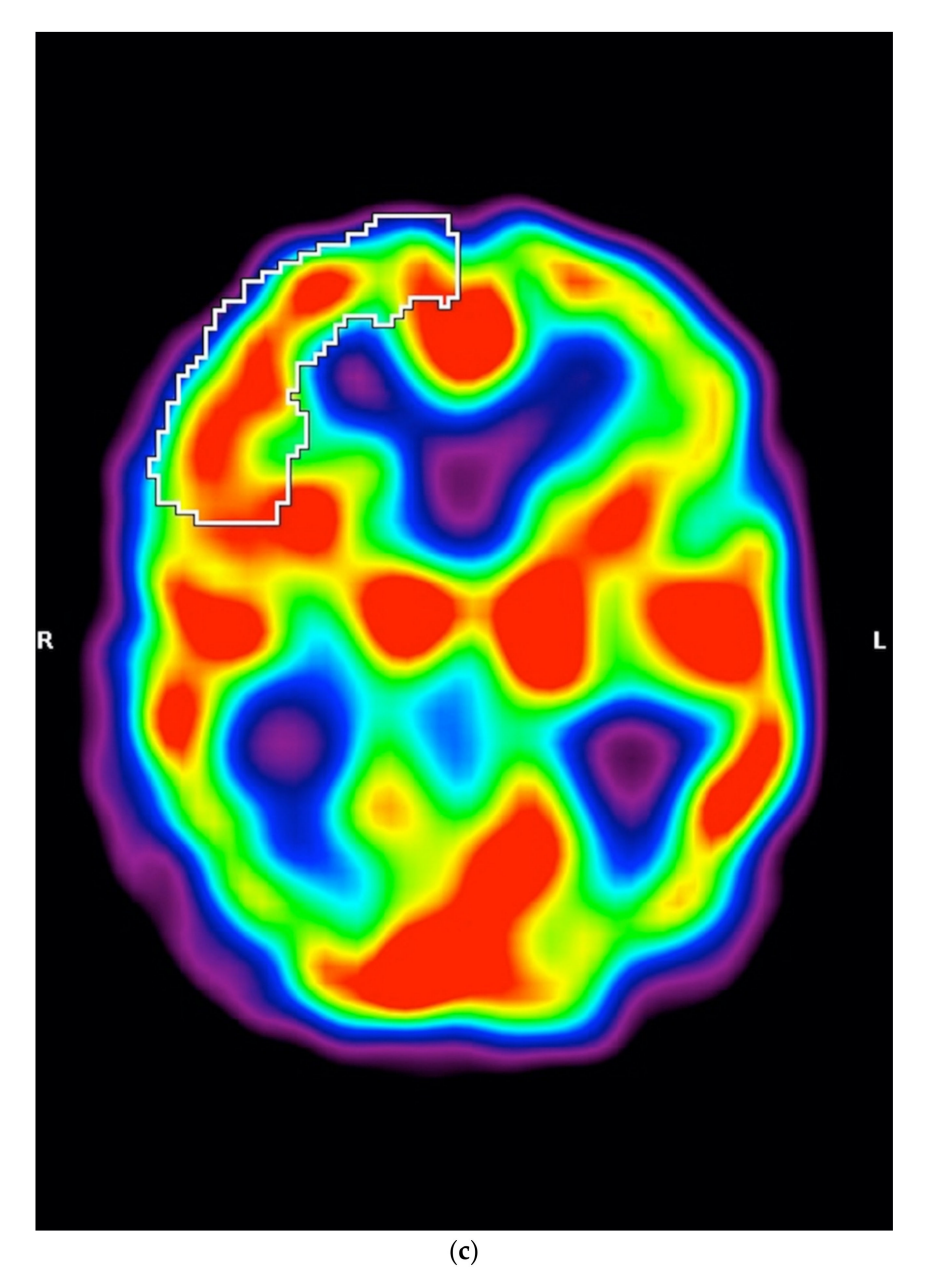
(**a)** Scatterplot presenting the significant differences between MSA-P and PSP-P in the right frontal lobe, (**b**) Axial 99mTc-HMPAO SPECT of a patient with PSP-P (frontal lobe hypoperfusion), (**c**) Axial 99mTc-HMPAO SPECT of a patient with MSA-P (without frontal lobe hypoperfusion).

In SPECT imaging, patients with MSA-P had higher regional cerebral blood flow in the right frontal lobe (*p* = 0.0012) (Figure 2a–c).

## 6. Discussion

Previous studies demonstrated the role of MRI in the examination of the entities in the early years of the disease. In this study, the authors evaluated the period between 3 and 6 years duration, during which the entities could be expected to be potentially differentiable, using easily accessible cognitive assessment screening. This observation may be interpreted as coming to the same conclusions as the study conducted by Jecmenica-Lukic et al., where the authors showed a more beneficial course for PSP-P when compared with MSA-P [29]. The authors compared the differentiating potential of MMSE and MoCA with perfusion SPECT and MRI. The results show that among patients who cannot undergo MRI, an assessment of frontal perfusion may be a beneficial additional examination. The assessment of mesencephalon and M/P ratio is a feature described in various studies; however, frontal lobe perfusion evaluation has not been broadly evaluated in PSP-P. In this study, the authors used the Bonferroni correction; however, before using it, additional parameters showed significant differences (threshold below 0.05) between PSP-P and MSA-P. Among them could be mentioned the perfusion on the left frontal lobe (*p* = 0.0121), the perfusion of the right thalamus (*p* = 0.0314), the width of the third ventricle (*p* = 0.048) and the width of MCP (*p* = 0.0045). After implementing the Bonferroni correction, the significance in these regions was not maintained. The authors believe that the regions should be additionally verified in larger groups and interpreted as probably significantly differentiating (Table 1 and Table 2).

This is a study based on widely accessible, non-specialist methods. The aim of the work was to base the results on methods which could be implemented in clinical practice, which excluded more advanced tools. Additionally, the groups examined in this study are relatively small, as they consist of 16 to 20 patients. Patients included in the study were clinically diagnosed with probable or possible PSP-P or MSA-P. As all of the patients were alive during the research, no neuropathological examinations were conducted.

No control group was assessed in this study. In SPECT, as in previous studies by our research group, the data were compared with a reference database comprising 99mTcHMPAO brain scans of 20 healthy volunteers with an age range of 64–86 years (males and females) [25]. The abnormalities of MRI were cross-referenced to the current literature and reference values used in the Department of Imaging Diagnostics, in which the study was performed [21,22,24]. The study was based on a convenient sample. The reliability of the results is bound by features related to the rarity of the diseases.

The results obtained highlight that PSP-P presents overlaps on various grounds in the differential diagnosis of other atypical parkinsonisms, even with a 3–6-year duration. Cognitive assessment screening did not provide sufficient data. Moreover, features such as moderate/weak response to levodopa treatment, dysautonomia and RBD were present in both groups. None of the patients showed typically pronounced oculomotor dysfunction characteristics for a 3–6-year duration of PSP-RS. The authors are aware of the fact that MoCA and MMSE do not highlight the deficits related to the frontal lobe or cerebellar region; however, the aim of the study was to evaluate screening methods rather than perform a cross-sectional neuropsychological examination. Moreover, earlier studies undertaken by the same group demonstrated that Frontal Assessment Battery shows limited feasibility in PSP-P, when compared with PSP-RS [25].

## 7. Conclusions

To the best of our knowledge this is the first study evaluating the differential diagnosis of PSP-P and MSA-P in perfusion SPECT. PSP-P is a difficult entity in the context of the differential diagnosis of atypical parkinsonisms. The study stresses the need for additional neuroimaging examination in PSP-P and MSA-P due to their overlaps in clinical development. Further analysis in the field of assessment of atypical parkinsonism, which could be implemented in clinical practice, is required.

## Figures and Tables

**Table 1:** Basic statistics for research group and subgroups.

Parameter	Whole Group		PSP-P (N = 16)		MSA-P (N = 20)	
	Mean (Min-Max)	SD ± 95% CI	Mean (Min-Max)	SD ± 95% CI	Mean (Min-Max)	SD ± 95% CI
Age	66.53 (50–81)	9.21 ± 7.47–12.02	71.44 (61–81)	6.88 ± 5.08–10.65	62.6 (50–81)	9.08 ± 6.91–13.26
Psychological tests:						
MMSE	27.82 (21–30)	2.32 ± 1.79–3.32	28.6 (26–30)	1.26 ± 0.87–2.31	27.17 (21–30)	2.82 ± 2–4.79
MoCA	23.4 (16–29)	3.64 ± 2.67–5.74	21.6 (20–24)	1.52 ± 0.91–4.36	24.3 (16–29)	4.11 ± 2.83–7.51
MRI Parameters:						
III ventricle (mm)	10.09 (6–16)	2.62 ± 2.11–3.45	11.14 (6–16)	2.48 ± 1.8–3.99	9.35 (6–14)	2.52 ± 1.92–3.68
Pons (cm2)	4.72 (3.05–5.79)	0.56 ± 0.45–0.75	4.79 (3.75–5.79)	0.61 ± 0.44–1.01	4.68 (3.05–5.55)	0.54 ± 0.41–0.79
Midbrain (cm^2^)	0.96 (0.57–1.5)	0.23 ± 0.18–0.3	0.76 (0.57–1.03)	0.12 ± 0.09–0.21	1.08 (0.76–1.5)	0.19 ± 0.15–0.28
M/P ratio	0.21 (0.13–0.34)	0.05 ± 0.04–0.07	0.16 (0.13–0.2)	0.02 ± 0.01–0.03	0.23 (0.16–0.34)	0.05 ± 0.04–0.07
MCP width (mm)	7.57 (5.3–9.3)	1.04 ± 0.84–1.37	8.15 (6.8–9.3)	0.76 ± 0.55–1.22	7.16 (5.3–8.9)	1.03 ± 0.78–1.5
SCP width (mm)	2.95 (1.5–3.7)	0.48 ± 0.39–0.63	2.84 (1.5–3.5)	0.58 ± 0.42–0.93	3.03 (2.4–3.7)	0.4 ± 0.3–0.58
MRPI	13.81 (6.33–33.43)	5.75 ± 4.62–7.61	18.75 (12.02–33.43)	5.81 ± 4.17–9.6	10.6 (6.33–17.15)	2.61 ± 1.99–3.81
SPECT parameters:						
Amygdala L	−0.65 (−3.3–3.1)	1.84 ± 1.49–2.39	−0.85 (−3.3–3.1)	1.69 ± 1.25–2.62	−0.49 (−3.3–2.8)	1.97 ± 1.5–2.88
Amygdala R	−0.75 (−3.4–4.1)	1.41 ± 1.14–1.84	−0.77 (−2.5–1.3)	1.14 ± 0.84–1.76	−0.74 (−3.4–4.1)	1.62 ± 1.23–2.37
Basal Ganglia L	−1.97 (−7.4–1.5)	1.95 ± 1.58–2.54	−1.89 (−5.3–1)	1.87 ± 1.38–2.9	−2.03 (−7.4–1.5)	2.05 ± 1.56–3
Basal Ganglia R	−1.62 (−5.3–2.1)	1.58 ± 1.28–2.06	−1.82 (−5.3–1)	1.54 ± 1.14–2.38	−1.46 (−4.5–2.1)	1.64 ± 1.24–2.39
Brainstem	−2.86 (−7–1.6)	2 ± 1.61–2.66	−3.04 (−7–0.4)	2.23 ± 1.62–3.59	−2.73 (−4.8–1.6)	1.86 ± 1.4–2.8
Cerebellum L	−2.13 (−10.4–1.4)	2.72 ± 2.21–3.55	−1.19 (−5.4–1.4)	1.92 ± 1.42–2.98	−2.89 (−10.4–1.1)	3.06 ± 2.33–4.47
Cerebellum R	−1.64 (−9.5–2.1)	2.79 ± 2.26–3.64	−1.27 (−5.3–1.1)	2.16 ± 1.59–3.34	−1.95 (−9.5–2.1)	3.23 ± 2.46–4.72
Frontal Lobe L	−0.09 (−4.8–3.4)	2.02 ± 1.63–2.65	−1.05 (−4.8–3.4)	2.39 ± 1.75–3.77	0.64 (−2.8–2.6)	1.34 ± 1.02–1.96
Frontal Lobe R	−0.14 (−4.6–3.1)	2.26 ± 1.83–2.97	−1.5 (−4.6–3.1)	2.36 ± 1.73–3.72	0.88 (−3.6–2.7)	1.59 ± 1.21–2.32
Hippocampus L	−1.78 (−4.8–1.4)	1.68 ± 1.36–2.19	−1.67 (−4.2–0.9)	1.44 ± 1.06–2.22	−1.88 (−4.8–1.4)	1.88 ± 1.43–2.74
Hippocampus R	−1.38 (−4.8–3)	1.78 ± 1.44–2.32	−1.26 (−4.8–1.7)	1.82 ± 1.34–2.81	−1.48 (−3.6–3)	1.79 ± 1.36–2.62
Insula L	−2.39 (−8.6–4)	2.95 ± 2.39–3.85	−2.45 (−7.1–4)	2.81 ± 2.08–4.35	−2.35 (−8.6–3.5)	3.13 ± 2.38–4.57
Insula R	−0.92 (−5.2–5.4)	2.27 ± 1.84–2.97	−1.01 (−3.2–5.4)	1.99 ± 1.47–3.08	−0.85 (−5.2–4.4)	2.53 ± 1.92–3.69
Pons	−2.73 (−5–−0.3)	1.34 ± 1.07–1.78	−2.79 (−5–−0.3)	1.45 ± 1.05–2.34	−2.68 (−4.9–−0.5)	1.28 ± 0.96–1.92
Temporal L	0.38 (−3.3–3.5)	1.65 ± 1.33–2.16	0.2 (−3.2–3.5)	1.46 ± 1.08–2.26	0.53 (−3.3–3.2)	1.82 ± 1.37–2.68
Temporal R	1.5 (−2.7–4.5)	1.63 ± 1.32–2.12	1.26 (−0.3–4)	1.21 ± 0.89–1.87	1.69 (−2.7–4.5)	1.91 ± 1.45–2.79
Thalamus L	−3.28 (−7–0.6)	1.8 ± 1.46–2.35	−3.71 (−7–−1.1)	2.08 ± 1.54–3.22	−2.94 (−5.3–0.6)	1.52 ± 1.15–2.22
Thalamus R	−3.36 (−7.6–1.5)	1.81 ± 1.47–2.36	−4.08 (−7.6–−1.7)	1.87 ± 1.38–2.9	−2.79 (−6.4–1.5)	1.58 ± 1.2–2.3
Whole Brain	−1.73 (−4.9–1.5)	1.63 ± 1.31–2.18	−2.13 (−4.9–1.5)	1.96 ± 1.42–3.15	−1.39 (−3–1.4)	1.28 ± 0.95–1.95

Green regions are feasible in the differential diagnosis of MSA-P and PSP-P; yellow regions require further research based on larger groups of patients and are probably feasible in the differential diagnosis of MSA-P and PSP-P.

**Table 2:** Subgroup comparison.

Parameter	*p*
Psychological tests:	
MMSE	0.154
MoCA	0.1851
MRI Parameters:	
III ventricle (mm)	0.0480
Pons (cm^2^)	0.5947
Midbrain (cm^2^)	0.0000
M/P ratio	0.0000
MCP width (mm)	0.0045
SCP width (mm)	0.2823
MRPI	0.0000
SPECT parameters:	
Amygdala L	0.5663
Amygdala R	0.9525
Basal Ganglia L	0.831
Basal Ganglia R	0.5065
Brainstem	0.6735
Cerebellum L	0.061
Cerebellum R	0.4776
Frontal Lobe L	0.0121
Frontal Lobe R	0.0012
Hippocampus L	0.7195
Hippocampus R	0.7274
Insula L	0.9173
Insula R	0.8349
Pons	0.8253
Temporal L	0.5673
Temporal R	0.4347
Thalamus L	0.2061
Thalamus R	0.0314
Whole Brain	0.2186

*p*-value for Student’s *t*-test; highlighted using green are statistically significant *p*-values after Bonferroni correction; highlighted using yellow are *p*-values below 0.05 (regions requiring further research based on larger groups of patients).

## Data Availability

The data presented in this study are available in the Supplementary Materials.

## Supplementary Materials

The following supporting information can be downloaded at: www.mdpi.com/article/10.3390/diagnostics12020385/s1, Table S1: General information regarding patients included to the study.
