# Supplementary material for: The Strengths and Obstacles in the Differential Diagnosis of Progressive Supranuclear Palsy—Parkinsonism Predominant (PSP-P) and Multiple System Atrophy (MSA) Using Magnetic Resonance Imaging (MRI) and Perfusion Single Photon Emission Computed Tomography (SPECT)
Source: Diagnostics (Basel). 2022 Feb 2;12(2):385. doi: 10.3390/diagnostics12020385 (PMC8871165; doi:10.3390/diagnostics12020385)
Supplement: Supplementary file 1 [file diagnostics-12-00385-s001.zip › diagnostics-1552308-supplementary.pdf]

Table S1. General information regarding patients included to the study.

Age	Gender	Diagnosis	Duration (YEARS)	Stage*
61	MALE	PSP-P	5	Advanced
74	FEMALE	PSP-P	3	Moderate
81	MALE	PSP-P	5	Advanced
80	FEMALE	PSP-P	4	Moderate
66	FEMALE	PSP-P	3	Moderate
72	FEMALE	PSP-P	5	Advanced
64	MALE	PSP-P	4	Moderate
76	MALE	PSP-P	5	Advanced
62	FEMALE	PSP-P	3	Moderate
69	FEMALE	PSP-P	3	Moderate
78	MALE	PSP-P	5	Advanced
77	FEMALE	PSP-P	3	Moderate
61	MALE	PSP-P	3	Moderate
75	FEMALE	PSP-P	4	Moderate
70	FEMALE	PSP-P	3	Moderate
77	FEMALE	PSP-P	5	Advanced
54	FEMALE	MSA-P	3	Moderate
67	FEMALE	MSA-P	6	Advanced
56	FEMALE	MSA-P	6	Advanced
72	FEMALE	MSA-P	3	Moderate
51	FEMALE	MSA-P	3	Moderate
69	MALE	MSA-P	4	Moderate
53	FEMALE	MSA-P	4	Moderate
64	MALE	MSA-P	5	Advanced
59	FEMALE	MSA-P	4	Moderate
73	MALE	MSA-P	4	Moderate
56	MALE	MSA-P	3	Moderate
56	FEMALE	MSA-P	4	Moderate
50	FEMALE	MSA-P	5	Advanced
74	FEMALE	MSA-P	3	Moderate
59	MALE	MSA-P	5	Advanced
75	FEMALE	MSA-P	6	Advanced
81	MALE	MSA-P	5	Advanced
55	FEMALE	MSA-P	3	Moderate
66	MALE	MSA-P	5	Advanced
62	FEMALE	MSA-P	4	Moderate

*) – stages: 1-2 years – EARLY, 3-4 years – MODERATE, 5 and above - ADVANCED
